# Comparative genomics of *Escherichia coli *isolated from patients with inflammatory bowel disease

**DOI:** 10.1186/1471-2164-12-316

**Published:** 2011-06-15

**Authors:** Rebecca Munk Vejborg, Viktoria Hancock, Andreas M Petersen, Karen A Krogfelt, Per Klemm

**Affiliations:** 1 Microbial Genomics and Antibiotic Resistance Group, DTU Food, Technical University of Denmark, DK-2800 Lyngby, Denmark; 2 Department of Gastroenterology, Hvidovre University Hospital, DK-2650 Hvidovre, Denmark; 3 Department of Bacteriology, Mycology and Parasitology, Statens Serum Institut, DK-2300 Copenhagen S, Denmark

**Keywords:** Inflammatory bowel disease, *Escherichia coli*, Urinary tract infections, LF82

## Abstract

**Background:**

Inflammatory bowel disease (IBD) is used to describe a state of idiopathic, chronic inflammation of the gastrointestinal tract. The two main phenotypes of IBD are Crohn's disease (CD) and ulcerative colitis (UC). The major cause of IBD-associated mortality is colorectal cancer. Although both host-genetic and exogenous factors have been found to be involved, the aetiology of IBD is still not well understood. In this study we characterized thirteen *Escherichia coli *strains from patients with IBD by comparative genomic hybridization employing a microarray based on 31 sequenced *E. coli *genomes from a wide range of commensal and pathogenic isolates.

**Results:**

The IBD isolates, obtained from patients with UC and CD, displayed remarkably heterogeneous genomic profiles with little or no evidence of group-specific determinants. No IBD-specific genes were evident when compared with the prototypic CD isolate, LF82, suggesting that the IBD-inducing effect of the strains is multifactorial. Several of the IBD isolates carried a number of extraintestinal pathogenic *E. coli *(ExPEC)-related virulence determinants such as the *pap*, *sfa*, *cdt *and *hly *genes. The isolates were also found to carry genes of ExPEC-associated genomic islands.

**Conclusions:**

Combined, these data suggest that *E. coli *isolates obtained from UC and CD patients represents a heterogeneous population of strains, with genomic profiles that are indistinguishable to those of ExPEC isolates. Our findings indicate that IBD-induction from *E. coli *strains is multifactorial and that a range of gene products may be involved in triggering the disease.

## Background

The term inflammatory bowel disease (IBD) is used to describe a state of idiopathic, chronic inflammation of the gastrointestinal tract. The two main phenotypes of IBD are Crohn's disease (CD) and ulcerative colitis (UC). CD is characterized by granulomatous inflammation and may affect any part of the gastrointestinal tract but is particularly prevalent in the ileocaecal area. UC, on the other hand, which is associated with extensive epithelial damage, crypt abscesses and significant neutrophil infiltration, is colon-specific. Patients suffering from extensive ulcerative colitis or colonic CD have a 10-fold increased risk of developing colorectal cancer, the major cause of IBD-associated mortality. The prevalence of IBD is 1-20 cases per 100,000 individuals and up to two million people are estimated to be affected by IBD worldwide [1,2]. IBD is more prevalent in developed countries than in developing countries, which has given rise to a number of theories regarding the significance of dietary preferences for the development and exacerbation of IBD [3-5].

Although the aetiology of IBD is still not well understood, it is generally believed that both host, environmental and microbial factors are involved. Several host-genetic factors (e.g. NOD2/CARD15) and exogenous factors (e.g. diet and smoking) have been identified [6]. Growing evidence also indicates that IBD results from an inappropriate immune response to the intestinal flora in genetically susceptible individuals. Animal models have shown that colitis does not occur in germ-free environment, but can be induced by the addition of bacteria [7,8]. The large intestine and colon are heavily colonized by microorganisms. While the bacteria associated with the intestinal flora are usually associated with a commensal lifestyle and form symbiotic multi-species communities, these bacteria may in some cases cause disease. There is mounting evidence that the composition of the microbiota in patients suffering from IBD is abnormal, and that the biodiversity is lower than in healthy subjects [9]. A range of bacterial species have been implicated in IBD, including *Escherichia coli*. Several studies have reported increased levels of *E. coli *in IBD tissues [10-12]. IBD patients have also been found to display increased serum immune-reactivity against *E. coli *as compared to healthy control subjects [13]. The identification of specific *E. coli *isolates with adherent and invasive capabilities in relation to CD patients has led to the coining of a new *E. coli *pathotype, the adherent-invasive *E. coli *(AIEC) [14]. The complete genome sequences of the prototypic CD *E. coli *isolate, LF82, as well as one other CD isolate, NRG857c, were recently published [15,16] and may pave the way for an increased understanding of the involvement of AIEC in IBD. Interestingly, studies with various probiotic microorganisms, including *E. coli *Nissle 1917, have shown that some probiotics may have a positive effect on inflammatory bowel disease [17-19]. This effect might be due to bacterial competition [20].

We have previously described the design and application of a 31-genome CGH microarray of *E. coli *[21,22]. In this study we have used this array to examine the genomic profile of a range of IBD isolates. We recently characterized a set of *E. coli *strains isolated from IBD patients [23]. Here we present a detailed comparative genomics study of these strains. We show that IBD isolates represents a heterogeneous population of strains, which display a remarkable resemblance with ExPEC isolates. Given that most ExPEC isolates originate from the intestinal tract, where they normally are present only in low numbers, it is conceivable that in IBD patients these clones are overrepresented.

## Results

### Genomic profiling of IBD-related *E. coli *isolates

**Figure 1:**
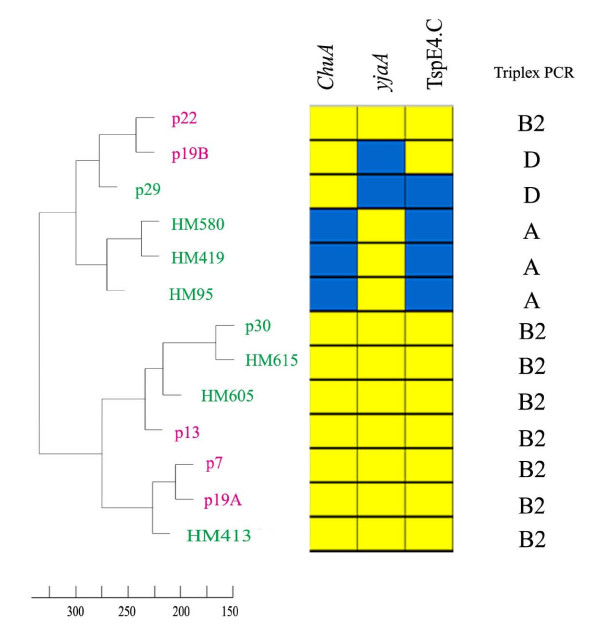
**Phylogenetic relationship between the isolates**. Relationship based on their overall genomic profiles (left side). The Crohn's disease isolates are indicated in green and the ulcerative colitis isolates in pink. CGH data for the three phylogroup identifiers *chuA*, *yjaA *and TspE4.C (middle). Phylogenetic group association based on triplex PCR data for the same identifiers (right side).

The comparative genomic analysis included five ulcerative colitis (UC) isolates, eight Crohn's disease (CD) isolates and the prototypic uropathogenic *E. coli *(UPEC) strain CFT073 (Table 1). Based on the CGH data, we initially sought to compare the IBD isolates with respect to their overall genomic profile. Interestingly, the comparison did not reveal the presence of two distinct subpopulations, i.e. UC and CD; rather it grouped the strains primarily according to their phylogenetic group (Figure 1). The only exception to this pattern was UC isolate p22, a group B2 isolate, which clustered most closely with UC isolate p19B, a group D strain. Therefore, based on the overall genotype, the UC and CD strains were indistinguishable. This clearly shows that the single most important determinant for the genomic profiles of IBD isolates is their phylogenetic group origin. With the exception of p22, this also suggests that the presence of horizontally acquired genetic segments does not generally obscure the phylogenetic signal. Within each phylogenetic group, some clustering according to disease was observed, although the relatively small number of isolates limits the delineation. Combined, the data shows that bacterial isolates from UC and CD display remarkably heterogeneous genomic profiles.

**Table 1:** Strains used in this study

*E. coli *strain	Relevant characteristics	Reference
Nissle 1917	Nonpathogenic probiotic isolate (O6:K5:H1)	[38]
MG1655	K-12 reference strain	[37]
LF82	Prototypic Crohn's disease isolate	[12]
p7	Ulcerative colitis isolate, active	[23]
p13	Ulcerative colitis isolate, active	[23]
p19A	Ulcerative colitis isolate, active	[23]
p19B	Ulcerative colitis isolate, active	[23]
p22	Ulcerative colitis isolate, active	[23]
p25	Ulcerative colitis isolate, active	[23]
p29	Crohn's disease isolate, active	[23]
p30	Crohn's disease isolate, active	[23]
HM95	Crohn's disease isolate	[36]
HM154	Crohn's disease isolate	[36]
HM413	Crohn's disease isolate	[36]
HM419	Crohn's disease isolate	[36]
HM580	Crohn's disease isolate	[36]
HM605	Crohn's disease isolate	[36]
HM615	Crohn's disease isolate	[36]
c1	Commensal isolate, O81:K16:H-	[23]
c2	Commensal isolate, O6:K39:H-	[23]
c3	Commensal isolate, O77:K96:H18	[23]
c4	Commensal isolate, O57, O155:K39:H19	[23]
c5	Commensal isolate, OX184:K-:H10	[23]
c6	Commensal isolate, O126:K-:H20	[23]
c14	Commensal isolate, Oru:K18:H19	[23]
c16	Commensal isolate, O1:K1:H-	[23]
c17	Commensal isolate, O101:K+:H56	[23]
p10A	Ulcerative colitis isolate, inactive eperioperiperiodperiod	[23]
p10B	Ulcerative colitis isolate, inactive	[23]
p23	Ulcerative colitis isolate, inactive	[23]
p26	Ulcerative colitis isolate, inactive	[23]
p27	Ulcerative colitis isolate, inactive	[23]
p32	Ulcerative colitis isolate, inactive	[23]
p11	Crohn's disease isolate, inactive	[23]
p15	Crohn's disease isolate, inactive	[23]
p31	Crohn's disease isolate, inactive	[23]

### IBD-specific markers

**Figure 2:**
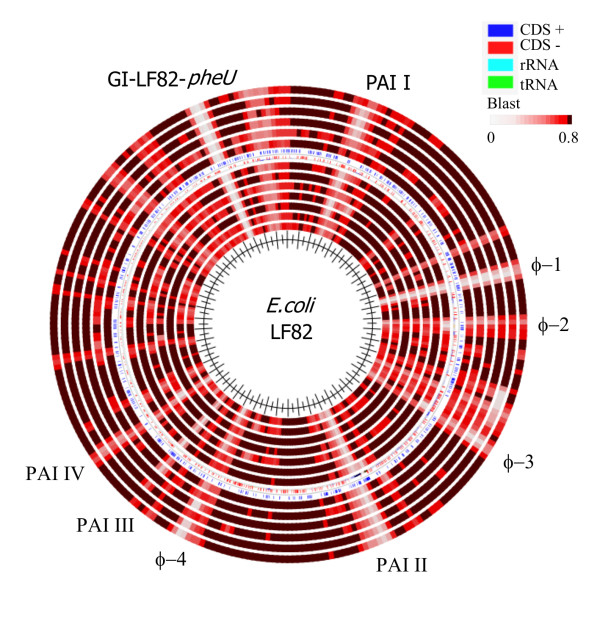
**Blast atlas comparison generated by blasting the inferred genomic sequences of the IBD isolates against the prototypic Crohn's disease isolate LF82 using the GeneWiz browser (**http://www.cbs.dtu.dk/services/gwBrowser/**)**. Blast lanes from the centre (eight CD isolates followed by five UC isolates): HM95, HM413, HM419, HM580, HM605, HM615, p29, p30, p7, p13, p19A, p19B and p22.

The implication of a specific *E. coli *pathovar (or pathovars) in the aetiology of IBD has remained elusive, mostly due to the lack of definable IBD marker genes. Recently, the complete genome sequences of two AIEC strains, i.e. LF82, a prototypic CD strain, and CD strain NRG857c, were published [15,16]. This may pave the way for a more systematic search for genetic determinants which can help to classify these pathogens. In order to look for commonalities between LF82 and the IBD isolates used in this study, we compared the inferred genomic sequence of our IBD strains with that of LF82. Initially this led us to examine the prevalence of LF82-associated genomic islands among the IBD isolates. Several of the genomic islands of LF82 appeared to be quite conserved among the IBDs, whereas others were less prevalent, or even LF82-specific (Figure 2). None of the isolates were found to carry to the GI-LF82-*pheU *island (mainly hypothetical proteins of unknown function) or the φ-1 prophage element, while most of the isolates were predicted to carry the high pathogenicity island (HPI), or PAI-II. The HPI island, which encodes yersiniabactin, is particularly prevalent among ExPEC isolates, and is known to contribute to the persistence of CFT073 in the urinary tract (murine model) [24]. The remaining islands were present in some, but not in all IBD isolates, clearly illustrating the heterogeneity of the population. Although each of these islands may contribute to the pathogenesis or fitness of LF82, most are clearly not descriptive of IBD isolates in general.

The complete genome sequence of LF82 allowed Miquel et al. to identify 115 LF82-specific genes by comparison with other sequenced *E. coli *genomes. However, the requirement or contribution of these genes to the pathogenesis of LF82 remains unknown, as does the general prevalence of these genes among IBD isolates. Of the 115 genes, 22 are shared by the other sequenced AIEC strain NRG857c. We analysed the CGH data to examine the prevalence of these genes among our IBD strains. Of the 115 LF82-specific genes, 57 were represented on the CGH microarray. Of these only seven were found present in one or more IBD strains examined, and none were present in all strains. Among the 22 genes, which were shared by NRG857c, only two genes were predicted present (and then only in a single isolate). This clearly reveals that these genes are most likely strain-specific rather than IBD-specific genes. Although some LF82-specific genes that were not present on our microarray might be prevalent among IBD isolates, our results suggest that it may be very difficult to identify IBD-specific genetic markers.

### Adhesins and biofilm-related genes

**Figure 3:**
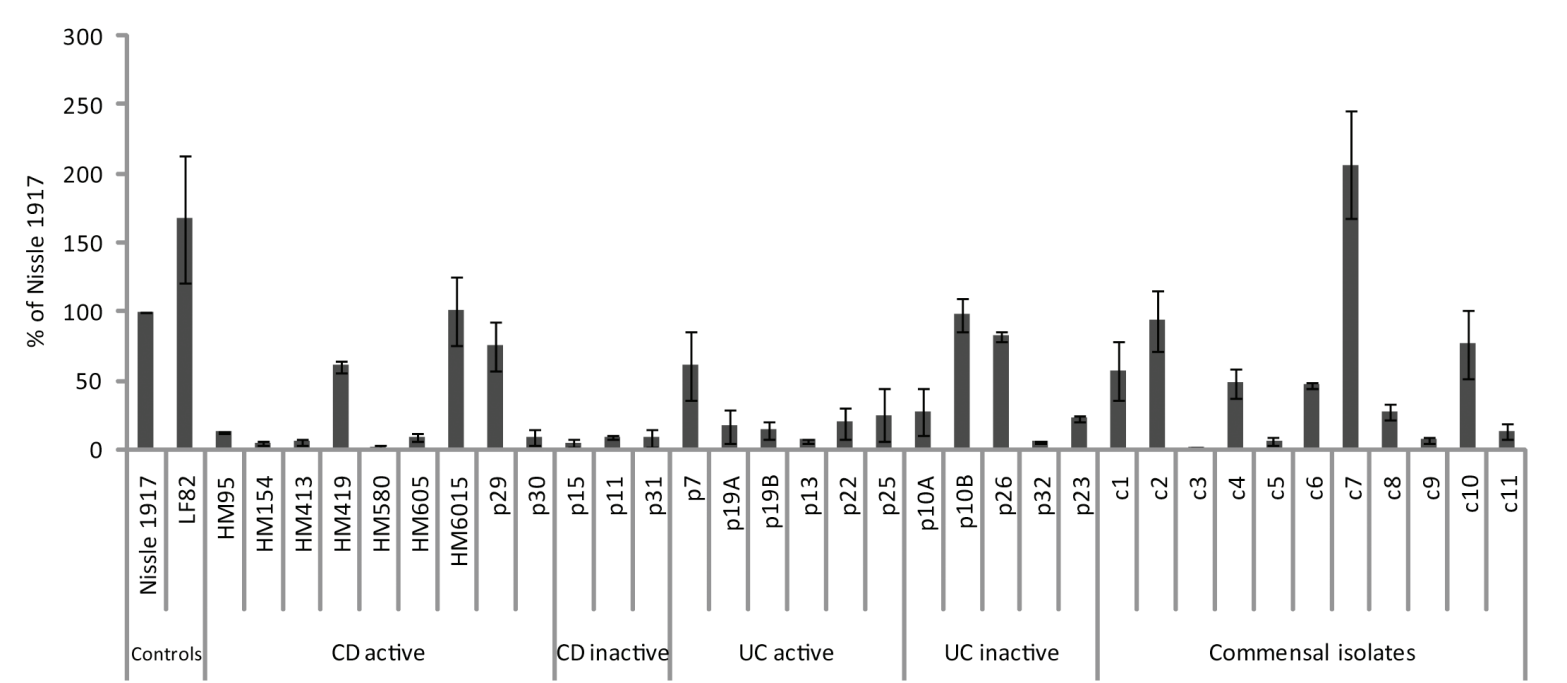
**Comparison of the biofilm-forming capabilities of the IBD isolates and *E. coli *isolates obtained from IBD patients during inactive periods, and healthy control persons**. Biofilm formation was monitored in LB medium using a crystal-violet, microtitre-based biofilm assay. Standard deviations are based on 3 independent experiments each comprising three replicates.

The ability to adhere to and colonize epithelial surfaces is a critical step in the development of many bacterial infections. Although the role of mucosa-associated *E. coli *in the development or exacerbation of IBD remains controversial, the association of *E. coli *isolates with the mucosal surface is intimately linked with the disorder(s). In order to determine the general adhesion and virulence potential of the IBD isolates, the genomic profiles were examined for a number of known and putative colonization and virulence factors. Several fimbrial operon genes were found among the isolates, which could contribute to adhesion of these strains (Table 2). Interestingly, the majority of the isolates were predicted to carry P-fimbria-related genes; also, two UC and five CD isolates showed positive haemagglutination (Table 3). Other adhesion-related genes identified among some of the isolates included the *sil *genes (adhesin for cattle colonization), *shlA *(autotransporter), a filamentous haemoagglutinin and *hek *(adhesion determinant) (Additional file 1, Table S1). The isolates were also found to carry a number of biofilm-related genes such as the *fim*, *flu *and *csg *genes encoding type 1 fimbriae, Ag43 (autotransporter protein) and curli, respectively (Table 3). There is mounting evidence that bacterial biofilms plays an important role in intestinal colonization and there is a growing interest in the role of biofilm formation in inflammatory bowel disease [25]. However, there was little if any indication that the IBD isolates displayed any consistently better biofilm-forming capacity *in vitro *in LB than isolates obtained from healthy control individuals (Figure 3). Several of the isolates also carried a number of genes related to invasion, including *ibeA*, *invA *and *tia *(Additional file 1, Table S1).

**Table 2:** Prevalence of fimbrial operons among the IBD isolates^c^

	UC (5)^a^	CD (8)^a^	MG1655	LF82	CFT073^b^
*sfm*	4	4	-	-	+
*ybg*	0	2	+	+	-
*ycb*	0	0	+	-	-
*yra*	0	3	+	-	-
*glt/yhc*	0	2	+	-	-
*yad*	2	1	-	+	+
*yag, matB*	5	8	+	+	+
*sfa/foc operon*	2	0	-	-	+
*F9, yde*	3	4	-	+	+
*yeh*	5	7	+	+	+
*yfc*	4	4	-	+	+
*pap*	1	4	-	-	+
*ygi*	2	4	-	+	+
*auf*	4	3	-	+	+
*fim operon*	5	6	+	+	+
*pix*	0	0	-	-	-
*CS1*	1	2	-	-	-
*F17-like fimbriae*	2	0	-	-	-
*CS12-like fimbriae*	0	0	-	-	-
*lpf/lpf2*	0	0	-	+	-
*bfp*	0	0	-	-	-

^a ^() total number of isolates included in the analysis

^b ^Same results were obtained by both CGH analysis and genome sequence inspection

^c ^Present/ absent calls are based on the entire fimbrial operons. Some fimbriae may however still be functional even in the absence of some fimbrial genes.

**Table 3:** Phenotypic and genotypic characteristics of the IBD strains

Strains	Source	Group	Pellicle	*flu*	*fim^a^*	*fimH^a^*	YA	*csg*	*pga*	CR^b^	Motility^c^	*pap*	HA^d^	*hly*	Haemolysis
MG1655	Commensal isolate	A	+	+	*BEAICDFGH+*	*+*	+	*CABDEFG+*	*ABCD+*	+	+	-	-	-	-
Nissle 1917	Probiotic strain	B2	+	+	*BEAICDFGH+*	*+*	+	*CABDEFG+*	*ABCD+*	++	+	*IA+*	-	*ABCD+*	-
CFT073	UPEC strain	B2	+	+	*BEAICDFGH+*	*+*	+	*CABDEFG+*	*ABCD+*	+	+	*IAHCDJKEFG+*	+	*ABCD+*	+
p7	Ulcerative colitis	B2	+	+	*BEAICDFGH+*	*+*	+	*CABDEFG+*	*ABCD+*	+	++	*IHCDJKFG+*	-	*ABCD+*	+
p13	Ulcerative colitis	B2	+	-	*BEAICDFGH+*	*+*	+	*CABDEFG+*	*ABCD+*	+	++	*-*	-	*B+*	-
p19A	Ulcerative colitis	B2	+	+	*BEAICDFGH+*	*+*	-	*CABDEFG+*	*ABCD+*	++	++	*IAHCDJKEFG+*	+	*ABCD+*	+
p19B	Ulcerative colitis	D	+	+	*BEAICDFGH+*	*+*	-	*CABDEFG+*	*ABCD+*	++	++	*IAEFG+*	-	*B+*	-
p22	Ulcerative colitis	B2	+	+	*BEAICDFGH+*	*+*	-	*CABDEFG+*	*ABCD+*	+	-	*IACDJKEFG+*	+	*ABCD+*	+
p29	Crohn's disease	A	+	+	*BEACDFGH+*	*+*	-	*CABDEG+*	*ABCD+*	++	++	*F+*	-	-	-
p30	Crohn's disease	B2	+	+	*BECDFGH+*	*+*	-	*CABDEFG+*	*ABCD+*	++	++	*IAHCDJKEFG+*	+	-	-
HM95	Crohn's disease	A	+	-	*BEAICDFGH+*	*+*	+	*CABDEFG+*	*ABCD+*	+++	++	*AF+*	-	*B+*	-
HM413	Crohn's disease	B2	+	+	*BEAICDFGH+*	*+*	+	*CABDEFG+*	*ABCD+*	+++	++	*-*	-	*B+*	-
HM419	Crohn's disease	A	+	+	*BEAICDGH+*	*+*	-	*CABDEG+*	*ABCD+*	+	-	*IA+*^f^	+^f^	-	-
HM580	Crohn's disease	D	+	+	*BEAICDFGH+*	*+*	-	*CABDEFG+*	*-^e^*	-	-	*IAHCDJKEFG+*	+	-	-
HM605	Crohn's disease	B2	+	-	*BEACDFGH+*	*+*	-	*CABDEG+*	*ABCD+*	++	-	*IAHCDJKEFG+*	+	-	-
HM615	Crohn's disease	B2	+	+	*BEAICDFGH+*	*+*	+	*CABDEFG+*	*ABCD+*	++	+	*IAHCDJKEFG+*	+	-	-

Abbreviations: YA: Yeast agglutination, HA: Haemagglutination

^a^Based on CGH data

^b^Congo-red binding

^c^Motility on LB plates, average of four plates. '-', non-motile; '+', average motility; '++', high motility (i.e. covered the whole plate, 80 mm)

^d^Same results were obtained with and without mannose

^e^Medium signal intensities were observed for this strain, i.e. that it cannot be excluded that it carries a divergent version of this operon

^f^The reason for this discrepancy remains unknown.

### Classical virulence genes

The IBD isolates did not carry any of the classical virulence determinants characteristic of intestinal pathogenic *E. coli *(Table 4). This correlates well with previous studies, which have also found little evidence of the involvement of specific types of intestinal *E. coli *pathotypes in the etiology of IBD [26]. However, several of the UC strains were found to carry the *hly *genes and displayed haemolytic activity (Table 3).

**Table 4:** Prevalence of various types of *E. coli *toxins and effectors among the IBD isolates

Virulence factor	Pathotype	UC (5)^a^	CD (8)^a^	Nissle 1917	CFT073
Shiga toxin I	EHEC	0	0	-	-
Shiga toxin 2	EHEC	0	0	-	-
EHEC haemolysin (*ehx*)	EHEC	0	0	-	-
Serine protease, EspP	EHEC	0	0	-	-
Serine protease, EspC	EPEC	0	0	-	-
Urease gene cluster	EHEC	0	0	-	-
Cell-cycle inhibiting factor (*cif*)	EPEC, EHEC	0	0	-	-
Type III secretion effector, Tir	EPEC, EHEC	0	0	-	-
Type III secretion effector, EspF	EPEC, EHEC	0	0	-	-
Type III secretion effector, EspH	EPEC, EHEC	0	0	-	-
Inhibition of lymphocytes activation (Lif/Efa)	EPEC, EHEC	0	0	-	-
Heat-labile enterotoxin (LT), alpha-subunit (*eltA*)	ETEC	0	0	-	-
Type III secretion effector, IpaB	EIEC	0	0	-	-
Shigella enterotoxin (ShET2)	EAEC, EIEC	0	0	-	-
Haemolysin *(hly)*	ExPEC	2	0	-	+
Cytotoxic necrotizing factor 1 (*cnf1*)	ExPEC, MNEC	2	0	-	-
Serine protease, Sat	ExPEC	4	2	+	+
Haemoglobin-binding protease (*tsh*)	ExPEC, APEC	0	0	-	-^b^
Cytolethal distending toxin (*cdt*)	Several	0	0	-	-

Abbreviations: Enterohaemorrhagic *E. coli *(EHEC), Enteropathogenic *E. coli *(EPEC), Enterotoxigenic *E. coli *(ETEC), Enteroinvasive *E. coli *(EIEC), Enteroaggregative *E. coli *(EAEC), Extra-intestinal pathogenic *E. coli *(ExPEC)

^a^() total number of isolates

^b^CFT073 carries a haemoglobin protease with homology to this, which is not represented on the chip

### Other virulence and fitness factors

In order to look for other potential genetic markers of IBD, we compared the inferred genomic profiles of the isolates with a range of sequenced commensal strains belonging to different phylogenetic groups. Initially we looked for genes that were present in all the IBD isolates but absent in the six commensal strains selected for the analysis (i.e. MG1655 (A), HS (A), IAI1 (B1), SE11 (B1), ED1a (B2) and SE15 (B2)). We also looked for genes common to all isolates within each disease category. No probes could specifically differentiate the UC and the CD isolates from the commensal strains. The UC isolates shared only one gene that was not present among the six commensal strains (i.e. *ECIAI39_1981*, encoding a hypothetical protein of bacteriophage origin). In CFT073, this gene is located in PAI-CFT073-*icdA*. Even within each phylogenetic group, only a few genes were conserved. Among the group A strains, only three genes were identified that were not present among the commensal group A strains, including two hypothetical proteins and a putative TonB-like protein. Among group B2, only the *ECIAI39_1981 *gene was identified. This clearly shows that the IBD isolates represent a heterogeneous population.

Given the heterogeneous nature of the genomic profiles, we proceeded to compare each individual isolate with the commensal strains (Additional file 1, Table S1). Except for the adhesin- and toxin-encoding genes already mentioned, several of the isolates were also predicted to carry lateral flagella. Interestingly, p22 was also found to carry the genes for lateral flagella, a trait which appears to be particular prevalent among group D strains. This may give some indication as to why this strain grouped with the D strains rather than the B2 strains (Figure 1). Several of the isolates were found to carry genes relating to propandiol utilization; however, none of the isolates were predicted to carry all of the *pdu *operon genes. Interestingly, several of the IBD isolates carried the *gipA *gene, a Peyer's patch-specific virulence factor, which is also present in LF82. A few isolates also carried *vapA*, coding for a putative virulence associated protein, as well as a range of ExPEC-related virulence genes (see subsequent section).

### UC- and CD-specific genes

The manifestations of UC and CD are distinctly different, as are the pro-inflammatory mediators seen in the two IBD phenotypes. We therefore decided to compare the genomic profiles of the two groups to see whether the *E. coli *isolates associated with UC and CD would display divergent genotypes. Overall, there were no genes that could entirely differentiate one group from the other, although a few probes were more associated with one group than the other (Additional file 2, Table S2). This clearly highlights the heterogeneity of the populations. It should be noted that isolates were not all obtained from the same origin, which could affect the analysis.

### Similarity to UTI isolates

**Figure 4:**
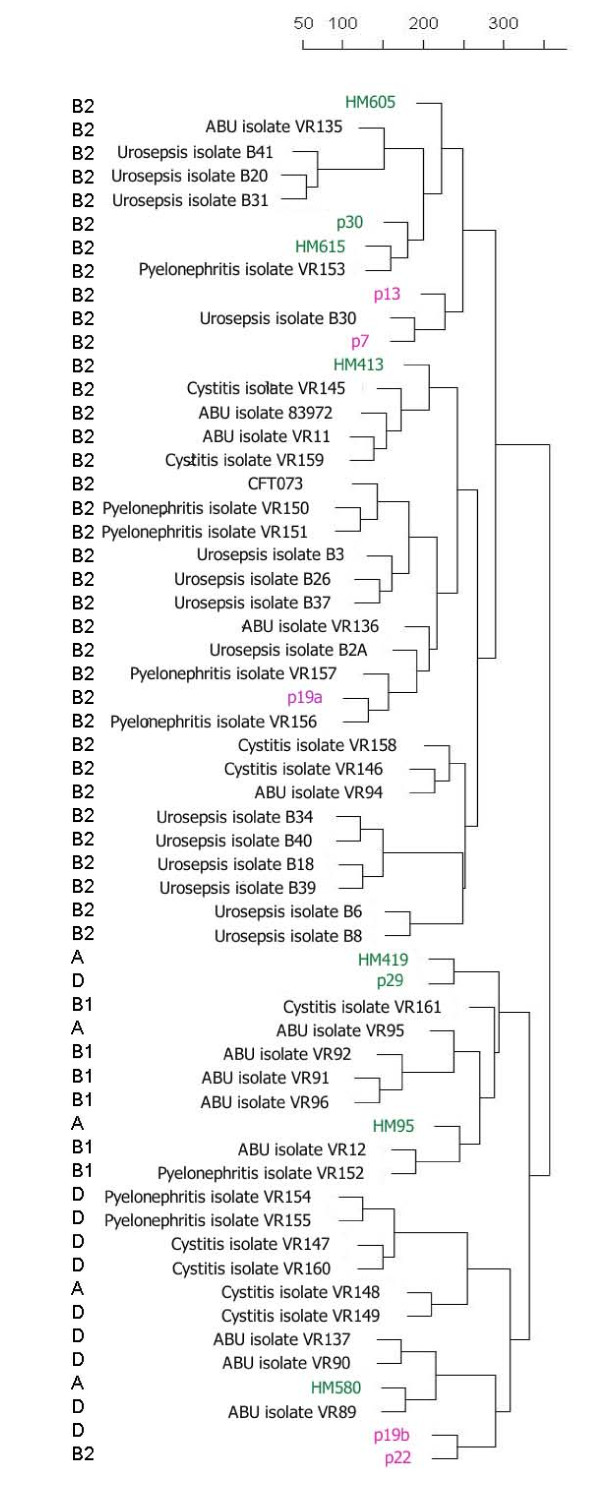
**Phylogenetic relationship between the IBD isolates and a range of UTI and urosepsis isolates**. The Crohn's disease isolates are indicated in green and the ulcerative colitis isolates in pink.

**Figure 5:**
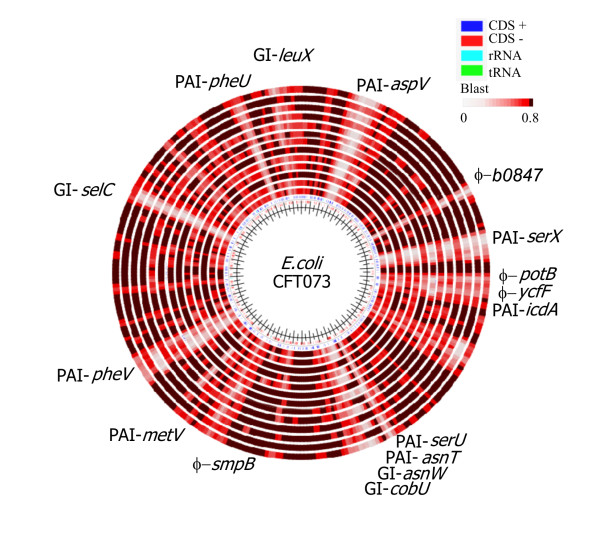
**Blast atlas comparison generated by blasting the inferred genomic sequences of the IBD isolates against the prototypic pyelonephritis isolate CFT073 using the GeneWiz browser service**. Blast lanes from the middle: HM95, HM413, HM419, HM580, HM605, HM615, p29, p30, p7, p13, p19A, p19B and p22.

Previous studies have shown that many IBD isolates carry a range of ExPEC-associated virulence determinants. Therefore, the IBD genomic profiles were compared with the CGH data obtained for a range of clinical ExPEC isolates (in this case UTI and urosepsis isolates). Based on their overall genomic profiles, there was no clear delineation between the various disease groups. The phylogenetic analysis grouped the isolates mainly according to their phylogenetic group, and little if any grouping of the IBD isolates was observed (Figure 4). We subsequently examined the prevalence of a range of CFT073-associated islands among the IBD isolates. CFT073 carries a number of well-characterized genomic islands [24], which are all represented on the CGH microarray. Interestingly, several of the IBD isolates were predicted to carry a number of these islands, some of which are not present in the sequenced commensal isolates, such as the CFT073 islands located at *aspV*, *serX*, *serU*, *asnW *(*pks*), *cobU*, *metV *and *leuX *(Figure 5). The *pks *island, encoding the machinery for the synthesis of colibactin (capable of inducing breaks in double-stranded DNA in eukaryotic cells), was present in three of five UC isolates, while only two of eight CD isolates carried this island. A few of the UC isolates were predicted to carry PAI-CFT073-*serU*, which have previously been shown to encode an immuno-modulatory protein TcpC, which is important for upper urinary tract infections [27,28]. Several of the IBD isolates also carried PAI-CFT073-*pheV *and PAI-CFT073-*pheU*, which encode P fimbriae and haemolysin; some strains were also found to express the phenotypes associated with these factors (Table 3). A few CD isolates carried GI-CFT073-*selC*. In accordance with the presence of a range of UPEC-associated islands, the IBD isolates were also predicted to carry and express a number of ExPEC virulence/fitness genes (Tables 2, 3 and 5). The presence of a number of CFT073-associated PAIs and expression of ExPEC virulence genes among the IBD strains clearly reveals that the IBD strains strongly resemble ExPEC strains.

**Table 5:** Prevalence of ExPEC virulence and fitness genes among the IBD isolates

Product	CFT073	p7	p13	p19A	p19B	p22	p29	p30	HM95	HM413	HM419	HM580	HM605	HM615
**Adhesins**														
F1C fimbriae	+	+/-	-	+	+/-	+	-	+/-	+/-	+/-	+/-	+/-	+/-	+/-
P fimbriae	+	+/-	-	+	+/-	+/-	+/-	+	+/-	-	+/-	+	+	+
Pix fimbriae	-	-	+/-	-	-	-	-	-	-	-	-	-	-	-
F17 fimbriae	-	+	-	+	-	-	-	-	+/-	-	-	-	-	-
CS12 fimbriae	-	-	-	-	-	-	-	-	-	-	-	-	-	-
*yapH*	+	+	+	+	-	-	-	-	+	+	-	-	-	-
ShlA/HecA/FhaA exoprotein family	+	+	+	+	-	+	-	-	+	+	-	-	-	-
**Toxins**														
RTX family exoprotein	+	+	-	+	-	-	-	-	-	+	-	-	-	-
Haemoglobin protease	+	+	+	+	-	+	-	+	-	+	-	-	+	+
*hly*	+	+	+/-	+	+/-	+/-	-	-	+/-	+/-	-	-	-	-
*sat*	+	+	-	+	+	+	+	-	-	-	-	+	-	-
*cdt*	-	-	-	-	-	-	-	-	-	+	-	-	-	-
*cnf1*	-	+	-	+	-	-	-	-	-	-	-	-	-	-
**Nutrition**														
*iro*	+	-	+	+	-	+	+/-	+/-	-	+	-	-	+	+
*pgt*	+	+/-	-	-	-	-	+/-	+/-	-	-	-	-	+	+
**Other**														
*tcpC*	+	-	-	+	-	-	-	-	-	-	-	-	-	-
*shiA homolog*	+	+	-	+	+	+	+	+	+	-	+	+	+	+
*pntC*	-	-	+	-	-	-	-	-	-	-	-	-	-	-
*ibeA*	-	-	+	-	-	-	-	-	-	-	-	-	-	-
K1 capsule genes	-	-	-	-	-	-	-	+	-	-	-	-	+	+
K2 capsule genes	+	+	-	+	-	-	-	-	-	-	-	-	-	-
K15 capsule genes	-	-	-	-	-	-	-	-	-	-	-	-	-	-
putative FMN-dependent dehydrogenase	-	+	-	+	-	-	-	-	-	-	-	-	-	-
PAI-CFT073-*asnW *(pks)	+	+	-	+	+/-	+	+/-	-	+/-	+	-	-	-	-
GimB island	-	-	-	-	-	-	-	+	-	-	-	-	+	+

+, - and +/- denotes presence, absence and partly present, respectively.

## Discussion

The data presented here shows that *E. coli *IBD isolates represent a heterogeneous population, and that the single most important determinant for the genomic profile of the isolates is their phylogenetic group origin. The data also suggests that it may be difficult to identify IBD-specific, or even CD- or UC-specific, genetic markers. This correlates well with previous reports of Crohn's disease and colon cancer isolates, which also showed that these isolates by no means represent uniform populations [29]. The results may also explain why the pursuit for IBD-specific genetic determinants among *E. coli *isolates essentially has remained fruitless. However, although no IBD-specific markers could be identified in this study (taking into account 57 of the 115 LF82-specific genes previously identified), it cannot be excluded that the LF82-specific genes that were not represented on the chip, or other *E. coli *genes missing on the chip, could be IBD-specific. As with many pathogens, virulence is often multifactorial and made up by an assortment of diverse virulence factors. The LF82-specific genes may therefore still prove to be important for the IBD-inducing capacity of that particular strain. Nevertheless, it indicates that it may be difficult if not impossible to find unique IBD genes. Our findings indicate that IBD-induction from *E. coli *strains is multifactorial and that a range of gene products can trigger the disease. The apparent lack of IBD- and group-specific genetic determinants among the isolates also suggests that the host-microbe interactions and the altered environment of the gut likely play the most important roles in the aetiology of IBD. Due to the predisposition of the patient, the bacterial colonization may invoke an autoimmune reaction, which escalates, leading to the clinical symptoms of IBD.

The prevalence of various adhesive structures among the isolates varied considerably. Only relatively few fimbriae-encoding genes were shared by all isolates, even within each disease category. Type I fimbriae has previously been shown to be important for the invasive and adhesive characteristic of the prototypic AIEC strain LF82 [30]. It targets the carcinoembryonic antigen-related cell adhesion molecule 6 (CEACAM6) that is expressed on the apical side of ileal epithelial cells [31]. The high level of ileal colonization that is observed in CD patients may be linked to an abnormal expression of CEACAM6, indicating the presence of predisposing factors [31]. Interestingly, while most of the IBD isolates examined in this study were found to carry the *fim *genes, far from all were able to agglutinate yeast. It therefore seems likely that some of the isolates achieve adherence be means of other surface structures. Interestingly, several strains were found to carry and express P-fimbriae. While usually associated with UTI strains, P fimbriae can promote intestinal colonization [32,33]. However, whether P fimbriae or any of the other adhesive structures carried by these IBD isolates contributes to adherence in the IBD-inflicted gut, remains to be determined.

In recent years, there has been a growing appreciation of the importance of biofilm formation in the development and exacerbation of a range of chronic infections. This prompted us to evaluate the biofilm formation capacity of our IBD isolates. Previous studies have suggested that AIEC strains may be more efficient biofilm producers than non-AIEC isolates [25]. However, in this study there was no indication that the IBD strains displayed superior biofilm capacities to the non-IBD control strains. There was also no correlation between the active or inactive state of the diseases, and the biofilm production displayed by the isolates. Experimental differences and strains variations (incl. demographic origin) may explain such discrepancy. We have also not focused specifically on AIEC strains, unlike the previous study.

Most of the IBD isolates studied here were found to carry, and express, a number of genetic markers that are associated with ExPEC isolates. ExPEC, which predominantly belong to the phylogenetic groups B2 and D, are generally believed to originate from the gut, where they represent a small fraction of the *E. coli *flora. It might be conceivable that ExPEC-like strains are overrepresented in the microbiota of IBD patients, possibly due to the altered gut environment caused by the underlying genetic and environmental factors. This correlates well with the finding that the levels of strains belonging to group B2 are increased in IBD patients as compared with control subjects [23]. *E. coli *strains belonging to group B2, and to some extent group D, are known to carry a number of virulence and fitness genes that are not present in strains belonging to groups A and B1. ExPEC strains are associated with a number of extraintestinal infections like urinary tract infections, meningitis, pneumonia and wound infections. ExPEC strains are also usually regarded as being incapable of intestinal infections [34,35]. Meanwhile, they are good long-term colonisers of the human gut and about 20% of *E. coli *gut isolates are ExPEC strains. Paradoxically, IBD-associated strains seem to be the only ExPEC-type strains that are associated with intestinal infections (such as CD and UC). Our data suggests that these strains are not genetically distinct from other ExPEC strains.

## Conclusions

Collectively, our findings indicate that IBD-associated *E.*coli represent a heterogeneous population of strains, whose genomic profiles greatly resembles that of ExPEC isolates. Arguably, our findings indicate that IBD-induction from *E. coli *is multifactorial and that different combinations of gene products may trigger the disease in a complex interplay with host parameters and environmental cues. Nevertheless, it still remains to be determined whether IBD-associated *E. coli *are directly involved in the pathogenesis of IBD or whether their presence is simply a result of the inflammatory response and the altered microbial environment of the IBD gut.

## Methods

### Bacterial strains and growth media

The strains used in this study are described in Table 1. HM95, HM154, HM413, HM419, HM580, HM605 and HM615 were kindly provided by Barry J. Campbell [36]. Two well-characterized *E.coli *strains, Nissle 1917 and MG1655, were included as controls in the phenotypic experiments [37,38]. All cultivations were performed in modified LB medium [39] or ABTG with 0.02% casamino acids. All strains were grown in modified LB medium prior to genomic DNA isolation.

### Microarray design and sample preparation

The detailed design of the CGH custom microarray has been described elsewhere [21]. The 31 *E. coli *genomes used for designing the microarray included the prototypic CD strain LF82 and several ExPEC isolates, such as uropathogenic and avian pathogenic *E. coli *strains, as well as other pathogenic *E. coli *strains (e.g. EHEC) and a number of K-12 isolates. The microarray consisted of 134,285 probe sets (of 50-75mers) representing 16,098 *E. coli *target genes. The genomic DNA was isolated using the "IllustraTM bacterial genomicPrep Spin Kit" (GE Healthcare, 28-9042-58), and the samples were diluted to the recommended concentration. Sample preparation was then carried out using the NimbleGen Arrays User's Guide for CGH analysis. All isolates, except the reference strain CFT073, were run as single samples. The supporting microarray data have been deposited in ArrayExpress (http://www.ebi.ac.uk/arrayexpress) with the accession numbers E-MEXP-3090, E-MEXP-3089 and E-MTAB-212.

### CGH data analysis

Data analysis was performed in R (statistical software), using the 'oligo' package for analysis of oligonucleotide arrays at the probe-level (Bioconductor) [40]. The RMA algorithm was used to perform background subtraction, normalisation and expression calculation (output in the log2 scale). Blast atlases were created using the GeneWiz whole genome visualization tool [41]. Hierarchical agglomerative clustering was performed in R, using the 'hclust' clustering method, an euclidean distance measure and the complete genomic profile of each strain (log values for all genes represented by more four or more probes). For interpretation of the CGH data, the following cut-off values (log values) were selected for presence/absence call of the individual probes: 6-8 negative, 8-10 borderline/unknown and 10-12 positive. The cut-off values were selected based on comparison of the CGH data obtained for CFT073 (run in triplicates) and the known genome sequence of this strain.

### Phylogenetic group determination

For identification of phylogenetic group associations, a triplex PCR method was employed, using primers targeting two genes (*chuA *and *yjaA*) and one anonymous DNA fragment (TspE4.C) [42].

### Statistical analysis

Statistical analysis was performed using Fischer's exact test when appropriate.

### Biofilm formation in microtitre plates

Cells were grown overnight in LB and used to inoculate flat-bottomed, non-treated 48-well plates (Nunc) to an OD_600 _of 0.05. The microtitre plates were incubated statically at 37°C for 16 hours, and the biofilm stained with 0.1% crystal violet for 15 min. Excess dye was removed by washing with PBS. Crystal violet was then solubilised by the addition of 96% ethanol and A_595 _was measured. All experiments were repeated at least three times.

### Yeast agglutination and haemagglutination tests

The presence of type 1 fimbriae was assayed by the ability of the bacteria to agglutinate yeast cells (*Saccharomyces cerevisiae*) on glass slides. Ten µl of fresh overnight cultures were mixed with 10 µl 5% yeast cells. The experiment was repeated after the cells had been washed and resuspended in LB containing 50 mM methyl-α-D-mannopyranoside.

The capacity of bacteria to express P fimbriae was assayed by haemagglutination with human type A red blood cells (RBCs). RBCs were washed twice with phosphate-buffered saline (PBS) and 10 µl of 5% RBCs were mixed with a single bacterial colony (freshly grown on LB plates) suspended in PBS on glass slides. Any strain showing positive haemagglutination was tested again after 30 min incubation with 1% D-mannose to rule out type 1 fimbriae and to further support that agglutination was likely mediated by P fimbriae.

### Motility on LB plates

One µl of overnight culture was stabbed into LB plates containing 0.3% agar. The distance of migration (the diameter of the ring around the inoculation site) was measured after 16 h of incubation at 37ºC. The assay was performed in duplicates and repeated twice.

### Haemolytic activity on blood agar plates

Isolated colonies were spot inoculated and production of haemolysin was detected by determining a zone of lysis under each colony on tryptone soya agar plates with sheep blood (Oxoid Deutschland GmbH) after overnight incubation of the tested strains. The assay was repeated independently twice.

### Congo Red-binding assay

The ability to express curli fimbriae was evaluated by streaking each strain on modified LB-agar plates (without NaCl) containing 0.004% Congo Red (CR) and 0.002% Coomassie Brilliant Blue G. CR binding was indicated by the presence of red or pink colonies after incubation overnight at 37°C. The assay was performed in duplicates and repeated independently twice.

## Authors' contributions

RMV planned, performed and analysed the CGH microarray data and drafted the manuscript. RMV and VH carried out the phenotypic experiments. RMV, VH and PK conceived and designed the study and wrote the manuscript. AMP provided strains and commented on the manuscript. KAK provided strains. All authors read and approved the final manuscript.

## Supplementary Material

Additional File 1 **Table S1**. **Tables presenting genes present in the different IBD isolates, but in none of the six commensal strains (MG1655, HS, IAI1, SE11, ED1a and SE15).** Click here for file

Additional File 2 **Table S2. List of probes that showed significant difference between the disease groups UC and CD**. Click here for file
